# Evaluating the Pediatric Behavior Guidance of Students Based on Actual Clinical Transcripts Scored by Faculty and Large Language Models: Pilot Comparative Study

**DOI:** 10.2196/83376

**Published:** 2026-06-12

**Authors:** Ishreen Kaur Dhillon, Gabriel Keng Yan Lee, Shijia Hu

**Affiliations:** 1 Faculty of Dentistry, National University of Singapore, 9 Lower Kent Ridge Road, Singapore, 119085, Singapore, 65 67727757, 65 67785742

**Keywords:** clinical mentoring, dental education, large language models, pediatric dentistry, dental students

## Abstract

**Background:**

Personalized feedback improves the clinical pediatric behavior guidance performance of students but is prohibitively time-consuming to provide. Large language models (LLMs) can automate the process of evaluating clinical sessions but are limited to text-only input and consistency issues.

**Objective:**

This study compared the use of text-only transcripts against the use of video recordings for evaluating the clinical behavior guidance performance of dental students. Additionally, the consistency and accuracy of LLMs in evaluating the transcripts were compared against a human assessor.

**Methods:**

This study was conducted by using 40 video-recorded clinical encounters involving final-year dental students who were managing patients aged between 4 and 12 years at the Faculty of Dentistry, National University of Singapore. The videos were scored by using a previously validated pediatric behavior guidance scale. Clinical encounters were transcribed verbatim and scored by a study member using a modified version of the scale (nonverbal components removed). The time taken to rate the transcripts was recorded. Video scores were compared with transcript scores. Both the free-to-use version and the paid version of the ChatGPT LLM were also used to score the transcripts; consistency was evaluated and compared against the human assessor.

**Results:**

The average time taken to rate the transcripts (mean 12, range 3-25 min) was significantly (*P*<.001) lower than the average video length (mean 73, range 37-120 min). Comparing transcript scores with video scores resulted in a consistency intraclass correlation coefficient of 0.830 (95% CI 0.679‐0.910; *P*<.001), demonstrating good reliability. Comparing transcript scores with the free-to-use LLM’s and paid LLM’s scores yielded an absolute agreement intraclass correlation coefficient of 0.729 (95% CI 0.475‐0.859; *P*<.001) and 0.670 (95% CI 0.377‐0.825; *P*<.001), respectively, demonstrating moderate agreement. The LLMs were inconsistent, producing variable scores with the same prompt. The free-to-use and paid versions produced the same score for all 3 runs in only 7 (18%) and 4 (10%) of the 40 clinical encounters, respectively.

**Conclusions:**

Using transcripts to evaluate students’ clinical behavior guidance was time-saving for faculty, demonstrated good agreement with video-based evaluation, and could improve clinical teaching. Although LLMs can automate the task, improvements are needed to improve their consistency and accuracy.

## Introduction

Managing pediatric dental patients is stressful for dental students and inexperienced practitioners, particularly when communicating with fearful and uncooperative children. Dental students experience 3 times the stress levels when compared to seasoned specialists [1]. Although lectures and seminars deliver theoretical knowledge, they are limited in terms of actual application [2]. Experiential learning during clinical sessions is essential for applying knowledge received from didactic teaching. Central to this process is guidance from faculty (ie, clinical supervisor), whose feedback helps to transform knowledge into clinical proficiency [3]. Real-time faculty feedback offers the greatest potential due to the immediacy and individualized feedback provided [4]. However, it is impossible to provide continuous instructional and coaching feedback for every student, as faculty often supervise multiple students during clinical sessions.

An alternative to real-time faculty feedback is the utilization of video-recorded clinical sessions, which have been shown to significantly improve dental students’ pediatric guidance scores in clinical situations [5]. Although video feedback enables faculty to evaluate the entire session for each student and provide feedback targeted at processes, it is prohibitively time-consuming. Each session can last up to 90 minutes, requiring full viewing before meaningful feedback can be developed and delivered.

The advancement of artificial intelligence (AI)–powered large language models (LLMs) allows for the evaluation of clinical interactions and generation of feedback on the improvement of pediatric behavior guidance, which can help to improve the process of teaching such guidance. Recently, an LLM was used to simulate patient-physician interactions for medical students; the clinical decision-making performance of the students who received feedback from the LLM significantly improved in subsequent patient-physician interactions when compared to that of students who did not receive any feedback [6]. In another study, an LLM (ie, a chatbot) provided feedback on medical students’ history-taking performance that was comparable to feedback from human assessors [7]. However, these studies were based on simulated clinical scenarios, with none examining the use of LLMs in actual clinical situations. The use of LLMs to provide feedback on behavior guidance in pediatric dentistry can address the issue of the extensive time requirement for providing personalized feedback based on video-recorded sessions—an area that has yet to be explored.

LLMs have inherent shortcomings that may limit their ability to evaluate clinical interactions. Currently, input is limited to only text, which does not consider other important facets of communication, including tone, physical gestures, and nonverbal cues. As such, an LLM’s ability to evaluate clinical interactions may not be wholly representative [8]. Furthermore, a recent study found that using a commercially available LLM to evaluate patient risk based on computer-simulated clinical data resulted in moderately correlated (*r*=0.605) risk scores and only 56% agreement on a diagnosis category, calling into question the consistency of LLMs in scoring clinical interactions [9].

The primary aim of this study was to determine if using text-only input was sufficient for evaluating pediatric behavior guidance performance in a clinical setting when compared to using video-recorded clinical interactions. The secondary aim was to examine the consistency and accuracy of commercially available LLMs in evaluating pediatric behavior guidance when compared against a human assessor.

## Methods

### Ethical Considerations

This study was approved by the National University of Singapore Institutional Review Board (NUS-IRB-2024‐934). This study was reported according to the Chatbot Assessment Reporting Tool (CHART) guidelines [10] (Checklist 1). Informed consent was obtained from participants. The participants were not provided with any monetary compensation. All data were deidentified before data analysis was done.

### Study Design

This study was conducted by using a set of 50 video-recorded clinical encounters involving final-year dental students who were managing pediatric patients aged between 4 and 12 years at the Faculty of Dentistry, National University of Singapore. The video-recorded clinical encounters were previously evaluated by a pediatric dentistry faculty member using a validated pediatric behavior guidance scale from an earlier study [5].

The video-recorded encounters were screened, and those not conducted primarily in English were excluded from this study (8 encounters). An accredited commercial company manually transcribed the videos and annotated the speakers to produce transcripts of the encounters for evaluation. Videos with audio that was too poor in quality for accurate transcription were excluded (2 encounters). A total of 40 encounters were successfully transcribed for this study. As this was a pilot study on using clinical transcripts to score behavior guidance, no sample size calculation was conducted.

**Figure 1.**
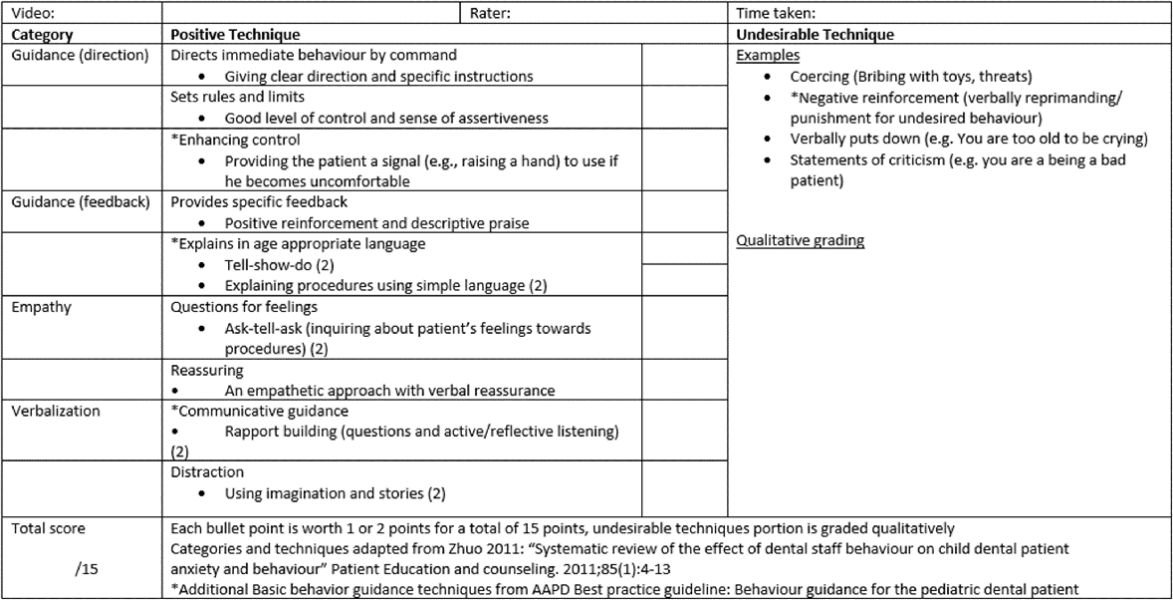
Modified rating scale for the rating of pediatric behavior guidance performance via the transcript (text-only) approach [11,12].

**Figure 2.**
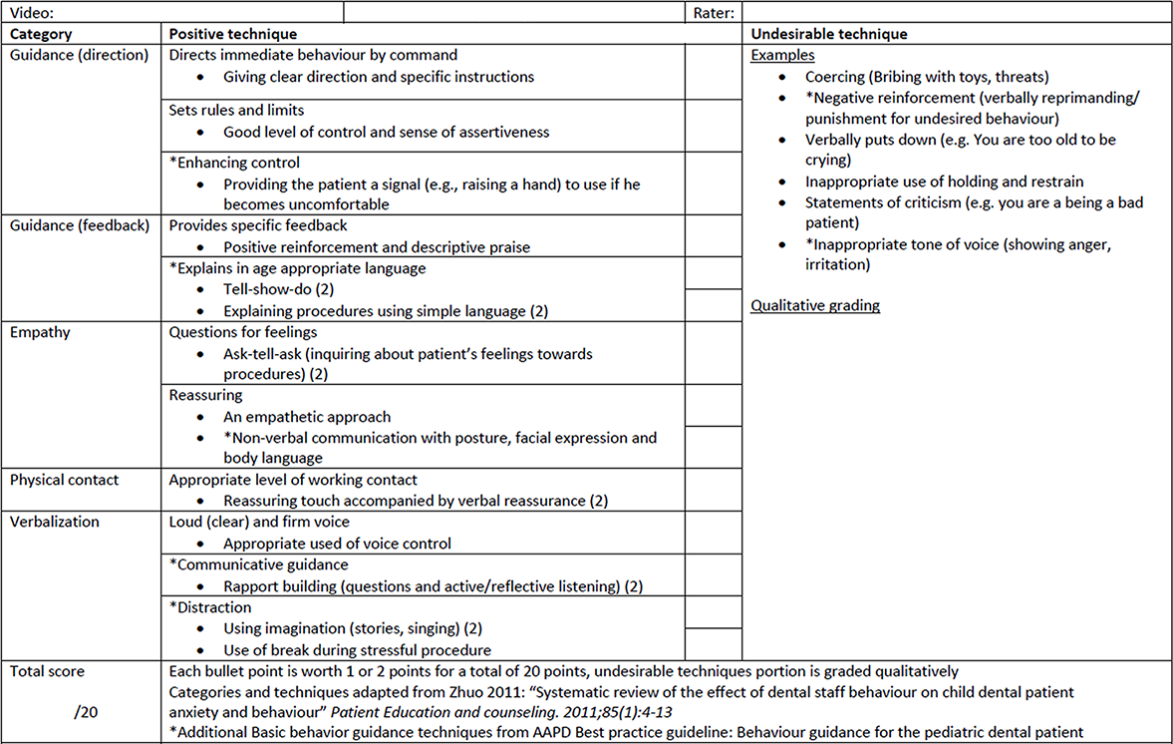
Original rating scale with nonverbal components [11,12].

A single study member (IKD) rated the transcripts by using a modified version of the previously validated scale [5], and the time taken by the human assessor to rate each encounter was recorded. The same study member randomly rerated 20% (8/40) of the transcripts, and the intrarater reliability bias–adjusted κ score was calculated to be 0.94. The pediatric behavior guidance scale was modified to remove nonverbal components, which are not captured with a text-only approach, giving the transcript (text-only) scale a maximum score of 15 (Figure 1), whereas the original scale had a maximum score of 20 (with higher scores denoting better clinical performance of behavior guidance techniques) in the original study (Figure 2) [5]. For comparison, the video scores from the original study were similarly modified to remove nonverbal components, resulting in a maximum possible score of 15. The transcript scores were compared to the video scores and the modified video scores without nonverbal components. This helped to determine if text-only information could be used to score clinical pediatric behavior guidance performance as reliably as clinical videos.

The free-to-use (GPT-4o) and paid (o4-mini-high) versions of ChatGPT (OpenAI) were guided with prompts (Multimedia Appendix 1) to rate behavior guidance performance by using the study rating scale and background information on pediatric behavior guidance (ie, from *The Reference Manual of Pediatric Dentistry* by the American Academy of Pediatric Dentistry) [5,11]. A clinical encounter transcript was uploaded to a new chat with the same prompt, and the score was recorded. This was repeated 3 times for each clinical encounter. The scores were marked as consistent if they matched in all 3 runs; if the scores were not consistent, the score with the higher frequency (matched in 2 out of 3 runs) or the middle score was used for the final analysis. Variability was reported as the average difference and the range of the difference between the highest and lowest scores for each encounter. The free-to-use LLM’s scores and the paid LLM’s scores were compared to the transcript scores to determine if LLMs could perform as well as humans when scoring behavior guidance performance.

Statistical analyses were conducted by using SPSS 29.0 statistical software (IBM Corporation). Descriptive statistics were used to report the consistency of LLM scoring, variability of LLM scores, length of videos, and time taken to rate transcripts. Comparisons were conducted by using the chi-square test and Wilcoxon signed rank test. Intraclass correlation (consistency) was conducted to compare video scores against modified video scores without nonverbal components and transcript scores to determine the reliability between the scales. Intraclass correlation (absolute agreement) and interclass correlation (Pearson coefficient correlation with bias adjustment) were conducted between transcript scores, modified video scores without nonverbal components, and LLM (free-to-use and paid versions) scores to determine the agreement of the scales.

## Results

A total of 40 clinical encounters were included in the final analysis. The average video length was 73 (range 37-120) minutes, and the average time taken to rate the transcripts was 12 (range 3-25) minutes. The time taken to rate the transcripts was significantly (*P*<.001) lower than the time taken to rate the videos.

The video scores were compared to the video scores without nonverbal components, resulting in a consistency intraclass correlation coefficient (ICC) of 0.978 (95% CI 0.959‐0.988; *P*<.001), which demonstrates excellent reliability.

The average video score (maximum=20) was 11.64 (SD 3.57), the average video score without nonverbal components (maximum=15) was 8.93 (SD 2.90), and the average transcript score (maximum=15) was 8.93 (SD 3.24). Comparing transcript scores to video scores resulted in a consistency ICC of 0.830 (95% CI 0.679‐0.910; *P*<.001), demonstrating good reliability, and comparing transcript scores to video scores without nonverbal components resulted in an absolute agreement ICC of 0.834 (95% CI 0.684‐0.912; *P*<.001), demonstrating good agreement (Table 1). The interclass correlation analysis showed similar results (Multimedia Appendix 2).

**Table 1.** Agreement of video scores, video scores without nonverbal components, and large language model (LLM) scores when compared against transcript scores.

	Average score (SD)	Compared with transcript scores, ICC^a^ (95% CI; *P* value)	Level of reliability/agreement^b^
Transcript scores (maximum=15)	8.93 (3.24)	—^c^	—
Video scores (maximum=20)	11.64 (3.57)	Consistency: 0.830 (0.679‐0.910; <.001)	Good
Video scores without nonverbal components (maximum=15)	8.93 (2.90)	Absolute agreement: 0.834 (0.684‐0.912; <.001)	Good
LLM (free-to-use) scores (maximum=15)	10.13 (3.32)	Absolute agreement: 0.729 (0.475‐0.859; <.001)	Moderate
LLM (paid) scores (maximum=15)	9.33 (3.14)	Absolute agreement: 0.670 (0.377‐0.825; <.001)	Moderate

a ICC: intraclass correlation coefficient.

b ICC ranges: above 0.90=excellent; between 0.75 and 0.90=good; between 0.50 and 0.75=moderate; below 0.50=poor [13].

c Not applicable.

The free-to-use LLM’s average score (maximum=15) was 10.13 (SD 3.32), and the paid LLM’s average score (maximum=15) was 9.33 (SD 3.14). Comparing transcript scores to the free-to-use LLM’s scores resulted in an absolute agreement ICC of 0.729 (95% CI 0.475‐0.859; *P*<.001), demonstrating moderate agreement, and comparing transcript scores to the paid LLM’s scores resulted in an absolute agreement ICC of 0.670 (95% CI 0.377‐0.825; *P*<.001), also demonstrating moderate agreement.

Both the free-to-use and paid versions of ChatGPT had low consistency, producing the same score in all 3 runs for only 7 out of 40 (18%) and 4 out of 40 (10%) clinical encounters, respectively. Additionally, the free-to-use version of ChatGPT had a significantly (*P*=.004) smaller average range of scores (mean 1.73, SD 1.18; range 0-4) when compared to that of the paid version of ChatGPT (mean 2.85, SD 1.70; range 0-7) (Table 2).

**Table 2.** Consistency and variability of the unpaid and paid versions of ChatGPT.

	Free-to-use ChatGPT (GPT-4o)	Paid ChatGPT (o4-mini-high)	*P* value
Consistency (among 3 runs with the same prompt for each clinical encounter [N=40])^a^, n (%)	7 (18)	4 (10)	.33
Variability (average difference for each encounter)^b^, mean (SD; range)	1.73 (1.18; 0-4)	2.85 (1.70; 0-7)	.004^c^

a Chi-square test.

b Wilcoxon signed rank test.

c *P*<.005.

## Discussion

This study found that using only transcripts to evaluate the clinical pediatric behavior guidance performance of dental students demonstrated good agreement when compared to using video evaluation. Moreover, transcripts required significantly less time to evaluate when compared to faculty evaluating full videos. Although the commercially available LLMs demonstrated moderate agreement with the faculty rater, they showed a lack of consistency, generating different scores under the same input parameters.

These findings have implications for resource optimization, as LLMs allow faculty to provide personalized feedback to more students, and the time saved allows for more clinical encounters per student. Adopting a theoretical framework of providing formative feedback continuously during training will have the greatest impact on student learning outcomes, which, in our case, was the improvement of clinical pediatric behavior guidance [14]. The use of LLMs can automate this task, thereby reducing the time burden on faculty. However, similar to a previous study, the commercially available LLMs in our study showed a lack of consistency, generating different scores under the same input parameters [9]. As the range of the LLM scores was relatively narrow and showed moderate agreement with the human assessor, future studies should evaluate if this lack of consistency would substantially alter the feedback provided or if the feedback could still be educationally useful. ChatGPT has been evaluated for other automated scoring applications, such as essay marking, with studies showing moderate to excellent correlation between ChatGPT and human assessors when using real-life datasets [15,16]. However, these studies also found wide task-dependent [15] and assessor-dependent [16] variation, and they cautioned the need for good rubric and prompt design and further LLM development before large-scale adoption.

A strength of this study was the use of real-life clinical encounters, as opposed to previous studies’ use of simulated clinical encounters [6,7,9,17]. Notably, the greater language variation present in the dataset could explain the LLMs’ large variation in scores and poor consistency. Further, while secondary refinement of a prompt after the first run improves the consistency of subsequent runs [18], this effect was lost when starting a new chat. This means that prompt engineering for every encounter or the use of an extremely complex and detailed prompt was necessary, making LLMs impractical for large-scale translation. Additionally, this study examined verbatim clinical encounter transcripts that contained informal language, such as slang and colloquialisms. Previous research showed that current LLMs have issues with processing informal language [19] and require substantial fine-tuning to achieve better performance [20]. This limitation in the LLMs used in this study possibly contributed to the large variation and poor consistency in scoring.

A limitation of this study was the use of only closed-source LLMs for the evaluation of pediatric behavior guidance. Both the free-to-use version and the paid version of ChatGPT resulted in similarly low consistency, with the free-to-use version demonstrating lower variability. It was difficult to speculate why the free-to-use version outperformed the paid version, as both were closed-source LLMs, but this warrants further investigation in future studies. Although closed-source LLMs provide several advantages, such as higher performance, easy implementation, and general accessibility due to significantly higher financial and technical backing, open-source LLMs are adaptable and cost-effective, thus allowing them to be customized and fine-tuned to specific domains, such as patient communication [21]. The transparency and inclusivity of open-source frameworks, such as Large Language Model Meta AI (LLaMA), could better address the specific requirements of dealing with the large variation and informal language present in real-life clinical communication datasets [22]. Future studies should compare the consistency and variability of closed-source and open-source LLMs.

In conclusion, using transcripts to evaluate students’ clinical behavior guidance was time-saving for the faculty and demonstrated good agreement with using videos for evaluation. Although LLMs can further automate the task, more work is needed to improve their consistency and accuracy. These findings can be used to improve the provision of targeted feedback to students for clinical teaching.

## Supplementary material

10.2196/83376 Multimedia Appendix 1 Prompts used to guide ChatGPT for the rating of pediatric behavior guidance performance.

10.2196/83376 Multimedia Appendix 2 Interclass (Pearson coefficient) correlation of transcript scores against video scores and video scores without nonverbal components.

10.2196/83376 Checklist 1 CHART checklist.

## ABBREVIATIONS

AI: artificial intelligence
CHART: Chatbot Assessment Reporting Tool
ICC: intraclass correlation coefficient
LLaMA: Large Language Model Meta AI
LLM: large language model
