# Supplementary figures and images for: Evaluating the Pediatric Behavior Guidance of Students Based on Actual Clinical Transcripts Scored by Faculty and Large Language Models: Pilot Comparative Study
Source: JMIR Med Educ. 2026 Jun 12;12:e83376. doi: 10.2196/83376 (PMC13263019; doi:10.2196/83376)

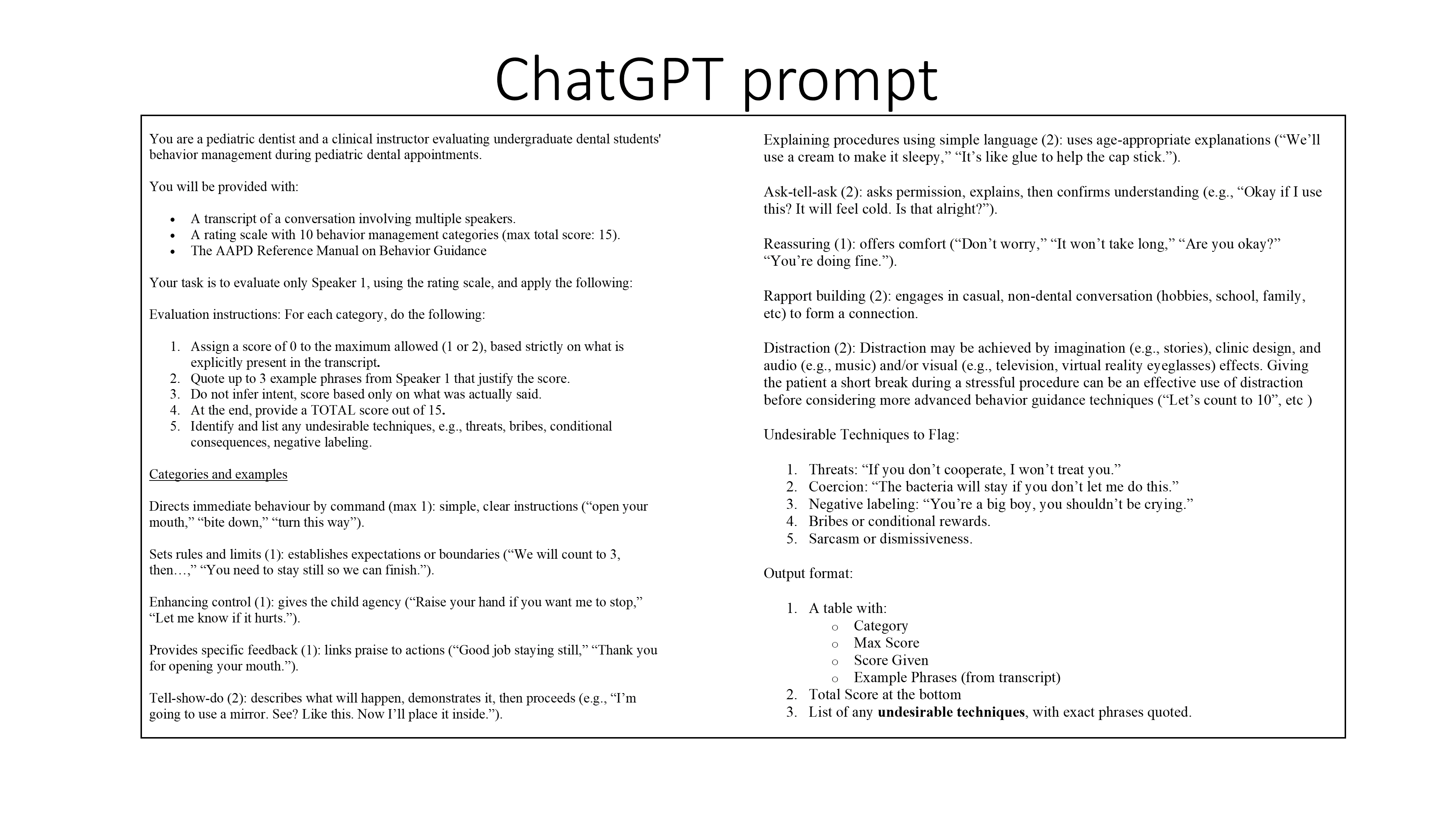

Supplement: Multimedia Appendix 1 [file mededu-v12-e83376-s001.png]
