# Supplementary material for: Evaluating the Pediatric Behavior Guidance of Students Based on Actual Clinical Transcripts Scored by Faculty and Large Language Models: Pilot Comparative Study
Source: JMIR Med Educ. 2026 Jun 12;12:e83376. doi: 10.2196/83376 (PMC13263019; doi:10.2196/83376)
Supplement: Multimedia Appendix 2 [file mededu-v12-e83376-s002.pdf]

	Average Score (SD)	Compared with transcript score Pearson correlation[a] (95% CI; *P* value)	Level of reliability/agreement
Transcript scores (maximum=15)	8.93 (3.24)	-	-
Video scores (maximum=20)	11.64 (3.57)	0.713 (0.509-0.835; *P*<.001)	Good
Video without non-verbal components (maximum=15)	8.93 (2.90)	0.714 (0.511-0.836; *P*<.001)	Good
LLM (free-to-use) scores (maximum=15)	10.13 (3.32)	0.606 (0.357-0.769; *P*<.001)	Moderate
LLM (paid) scores (maximum=15)	9.33 (3.14)	0.502 (0.219-0.700; *P*<.001)	Moderate
[a]With bias adjustment			
