# Supplementary material for: Evaluating the Pediatric Behavior Guidance of Students Based on Actual Clinical Transcripts Scored by Faculty and Large Language Models: Pilot Comparative Study
Source: JMIR Med Educ. 2026 Jun 12;12:e83376. doi: 10.2196/83376 (PMC13263019; doi:10.2196/83376)
Supplement: Checklist 1 [file mededu-v12-e83376-s003.pdf]

The CHART Checklist

HEADING	#	CHART CHECKLIST ITEM	Page #*
**Title & Abstract**			
**Title**	**1a**	State that the study is assessing one or more generative AI-driven chatbots for clinical evidence or health advice.	1
**Abstract/Summary**	**1b**	Apply a structured format, if applicable.	1
**Introduction**			
**Background**	**2a**	State the scientific background, rationale, and healthcare context for evaluating the generative AI-driven chatbot(s), referencing relevant literature when applicable.	2
	**2b**	State the aims and research questions including the target audience, intervention, comparator(s), and outcome(s).	2
**Methods**			
**Model Identifiers**	**3a**	State the name and version identifier(s) of the generative AI model(s) and chatbot(s) under evaluation, as well as their date of release or last update.	4
	**3b**	State whether the generative AI model(s) and chatbot(s) are open-source or closed-source/proprietary.	4
**Model Details**	**4a**	State whether the generative AI model was a base model or a novel base model, tuned model, or fine-tuned model.	4
	**4b**	If a base model is used, cite its development in sufficient detail to identify the model.	4
	**4c**	If a novel base model, tuned model, or fine-tuned model is used, describe the pre- and/or post-implementation/deployment data and parameters.	NA
**Prompt Engineering**	**5a**	Describe the evolution of study prompt development.	App1
	**5ai**	Describe the sources of prompts.	App1
	**5aii**	State the number and characteristics of the individual(s) involved in prompt engineering.	4
	**5aiii**	Provide details of any patient and public involvement during prompt engineering.	NA
	**5b**	Provide study prompts.	App1
**Query Strategy**	**6a**	State route of access to generative AI model.	4
	**6b**	State the date(s) and location(s) of queries for the generative AI-driven chatbot(s) including the day, month, and year as well as city and country.	NA
	**6c**	Describe whether prompts were input into separate chat session(s).	4
	**6d**	Provide all generative AI-driven chatbot output/responses	NA
**Performance Evaluation**	**7a**	Define the ground truth or reference standard used to define successful generative AI-driven chatbot performance.	4
	**7b**	Describe the process undertaken for generative AI-driven chatbot performance evaluation.	4
	**7bi**	State the number and characteristics of team members involved in performance evaluation.	4
	**7bii**	Provide details of any patients and public involvement during the evaluation process.	NA
	**7biii**	State whether evaluators were blinded to the identity of the generative AI-driven chatbot(s) under assessment.	NA

**Sample Size**	**8**	Report how the sample size was determined.	2
**Data Analysis**	**9a**	Describe statistical analysis methods, including any evaluation of reproducibility of generative AI-driven chatbot responses.	4
	**9ai**	Report the measures used for performance evaluation.	4
**Results**			
	**10a**	Report the alignment between generative AI-driven chatbot output and ground truth or reference standard using quantitative or mixed methods approaches as applicable.	4
	**10b**	For responses deviating from the ground truth or reference standard, state the nature of the difference(s).	4
	**10c**	Report the assessment for potentially harmful, biased, or misleading responses.	NA
**Discussion**			
	**11a**	Interpret study findings in the context of relevant evidence.	5
	**11b**	Describe the strengths and limitations of the study.	5
	**11c**	Describe the potential implications for practice, education, policy, regulation, and research.	5
**Open Science**			
**Disclosures**	**12a**	Report any relevant conflicts of interest for all authors.	6
**Funding**	**12b**	Report sources of funding and their role in the conduct and reporting of the study.	6
**Ethics**	**12c**	Describe the process undertaken for ethical approval.	2
	**12ci**	Describe the measures taken to safeguard data privacy of patient health information, as applicable.	2
	**12cii**	State whether permission/licensing was obtained for the use of original, copyrighted data.	NA
**Protocol**	**12d**	Provide a study protocol.	2-4
**Data availability**	**12e**	State where study data, code repository, and model parameters can be accessed.	6

*If in supplementary appendix, indicate “supp” and appendix #, if applicable.
